# Diet Quality and Risk of SARS-CoV-2 Infection or COVID-19: A Systematic Review of Observational Studies

**DOI:** 10.1016/j.advnut.2023.09.006

**Published:** 2023-09-23

**Authors:** Sukshma Sharma, Augusto Di Castelnuovo, Chiara Cerletti, Maria Benedetta Donati, Giovanni de Gaetano, Licia Iacoviello, Marialaura Bonaccio

**Affiliations:** 1 Department of Epidemiology and Prevention, IRCCS NEUROMED, Pozzilli (IS), Italy; 2 Mediterranea Cardiocentro, Napoli, Italy; 3 Research Center in Epidemiology and Preventive Medicine - EPIMED, Department of Medicine and Surgery, University of Insubria, Varese, Italy

**Keywords:** diet quality, SARS-CoV-2 infection, COVID-19, systematic review

## Abstract

The COVID-19 pandemic highlighted the importance of healthy diets in the management of severe acute respiratory syndrome coronavirus 2 (SARS-CoV-2) infection and COVID-19. Evidence suggests the influence of diet and dietary patterns during post–COVID-19, and the impact of the COVID-19 pandemic on dietary habits and quality. However, limited evidence lies on the association between a healthy diet, and risk of SARS-CoV-2 infection or COVID-19. This study aimed to conduct a systematic review of observational studies to examine the association between diet quality, and the risk of SARS-CoV-2 infection or COVID-19 among adult populations. 6158 research articles from Scopus, EMBASE, PubMed, and MEDLINE databases were identified for eligibility. Only observational studies were included. Study quality was assessed using the National Institutes of Health Quality Assessment Tool for Observational Cohort and Cross-Sectional Studies. Thirteen studies were included (4 with SARS-CoV-2 infection and 9 with COVID-19 as the outcome); 3 were case-control, 3 were cross-sectional, and 7 were prospective studies. Adherence to Mediterranean diet was examined as exposure in 7 studies, and was associated with decreased risk of SARS-CoV-2 infection in 2 studies, with estimates varying from 12% to 22%, while COVID-19 risk or severity was found to be reduced in 3 studies with odds ratios (ORs) ranging from 36% to 77%. The Dietary Approaches to Stop Hypertension diet was inversely associated with COVID-19 hospitalization (OR: 0.19; 95% CI: 0.07, 0.55), whereas a healthy plant-based diet had an inverse association with both COVID-19 infection (hazard ratio [HR]: 0.91; 95% CI: 0.88, 0.94) and severity (HR: 0.59; 95% CI: 0.47, 0.74). Studies examining individual food groups generally found lower risk of infection or COVID-19 in association with larger dietary intakes of fruits, vegetables, and fiber. The overall findings of the observational studies in this review support the concept that nutritious diets might lower the risk of SARS-CoV-2 infection or COVID-19.

This study was registered at PROSPERO as CRD42023397371.

**Statement of significance:**

1. To our knowledge, this is the first systematic review that investigated the association between diet and severe acute respiratory syndrome coronavirus 2 (SARS-CoV-2) infection or COVID-19 among observational studies. These results elucidate the importance of a wholesome diet that is beneficial in potentially protecting against SARS-CoV-2 infection or COVID-19.
2. This systematic review highlights the scarcity of observational studies that could be of public health importance and for cautious nutritional advice in clinical settings.

## Introduction

During the past 3 y, the world has been dealing with the Coronavirus Disease 19 (COVID-19) pandemic caused by a strain of coronavirus called the severe acute respiratory syndrome coronavirus 2 (SARS-CoV-2) [1,2]. Inflammation caused by the cytokine storm among COVID-19 infected cases is the result of a weak or overreactive immune system response [2,3]. Immunity levels among subjects are influenced by a variety of factors, including stress, physical activity, genetics, vaccination status, comorbidities, such as cardiovascular disease or metabolic syndrome, and diet (nutrition status) [4]. Among the mechanisms that are universally attributed to the effects of diet on diseases and health, diet-responsive effectors, specifically, the diet-immune axis, play an important role—for example, low protein can reduce the immunity status due to low antibody production [5,6]. Optimal nutrition is required to maintain the inter-relationship between immune system and modulated inflammatory and oxidative stress processes [7]. Known dietary and nutrient constituents that have anti-inflammatory and antioxidant properties are vitamin C [8], vitamin A [9], omega-3 fatty acids [10], and dietary fiber [11]. Further, diets rich in phytochemicals, such as polyphenols and fiber, are suggested to act as prebiotics, eventually promoting healthy bacterial growth, including *Bifidobacterium* species, which reduces diarrhea, a common symptom in SARS-CoV-2 infection and COVID-19 cases [12,13].

Among evidence related to diet, few studies have also explored the association between individual foods, and the risk of SARS-CoV-2 infection or COVID-19, including dragonfruit, flax seeds, basil, cinnamon, and ginger [[14], [15], [16]]. Further, studies have explored an inverse association between micronutrients, including vitamins (B_12_, C, and D) and minerals (iron, zinc, copper, and selenium), and the risk of COVID-19 [[17], [18], [19], [20]]. However, little is known regarding the associations between diet and the risk of SARS-CoV-2 infection or COVID-19 among observational studies among adult populations.

Therefore, the aim of this study was to conduct a systematic review of observational studies to examine the association between diet quality and the risk of SARS-CoV-2 infection or COVID-19 among adult populations.

## Methods

### Protocol registration

This systematic review was performed according to the PRISMA guidelines [21] (see the PRISMA checklist in Supplementary Table 2) and was registered at PROSPERO (reference CRD42023397371).

### Information sources and search strategy

A systematic search was conducted in December, 2022 and repeated in July, 2023 among 4 databases (EMBASE, PubMed, Medline, and Scopus), and no date restrictions were applied. Searches were conducted using predefined key words relating to dietary patterns, diets, dietary intakes, COVID-19, and SARS-CoV-2 infection with MeSH terms wherever applicable, such as “Mediterranean diet,” AND/OR “Mediterranean dietary pattern,” “Diet,” AND/OR “Diets,” “Dietary intake,” AND/OR “Dietary patterns,” AND “COVID-19” AND/OR “COVID-19” AND/OR “SARS-CoV-2 infection” (see Supplementary Table 3 for the full search strategy conducted within each database).

The reference lists of all articles were searched for potential studies to be included in the review. The full text versions were stored as PDF files, and all the studies extracted were managed and stored using Rayyan Software (an automated tool for systematic reviews) [22] and Mendeley Reference Manager version 2.79.0.

### Eligibility criteria

This systematic review only included observational studies (prospective cohort, case-control, and cross-sectional studies) that explored the association between diet quality (exposure) and risk of SARS-CoV-2 infection or COVID-19 (outcomes). Studies that examined severity and hospitalization because of COVID-19 were also included. Further, studies with adult human participants (>18 y) and examining dietary patterns based on food groups or components as the primary exposure, for example, a Mediterranean diet or “Western” dietary pattern, were included. Finally, studies that examined diets based on energy composition, for example, high-energy, low-fat, or high-protein diets, were also included.

The following studies were excluded: abstract, systematic review articles, editorials, case reports, letters, surveys, literature reviews, conference papers, thesis files, randomized controlled trials, and studies not conducted on human subjects. Also, studies that explored the influence of a single nutrient or food item, for example, vitamin C, iron, or ginger, on the risk of COVID-19 or SARS-CoV-2 infection were excluded. Finally, studies that were published in non-English language were also excluded.

### Definition of outcomes

As all the included studies in this systematic review used different methods for ascertainment of SARS-CoV-2 infection and COVID-19, we defined the 2 outcomes for the systematic review according to the NIH COVID-19 Treatment Guidelines (CTG) [23], mainly classified into the following 5 levels of severity: *1*) asymptomatic or presymptomatic infection: individuals with a positive test for SARS-CoV-2 using either a nucleic acid amplification test (includes RT-PCR tests along with Abbott ID NOW molecular rapid tests) or an antigen test but with no COVID-19 manifestations; *2*) mild illness: patients with any of the COVID-19 symptoms (e.g., loss of taste and smell, fever, headache, malaise, myalgia, nausea, vomiting, diarrhea, cough, and sore throat) but no dyspnea or abnormal chest imaging; *3*) moderate illness: individuals with clinical evidence of lower respiratory tract involvement or chest imaging and an oxygen saturation (SpO_2_) of ≥94% on room air at sea level; *4*) severe illness: individuals with a SpO_2_ <94% on room air at sea level, a partial pressure of oxygen/fraction of inspired oxygen ratio <300 mmHg, a respiratory rate >30 breaths/min, or lung infiltrates >50%; and *5*) critical illness: individuals suffering from respiratory failure, septic shock, and/or multiple organ dysfunction.

COVID-19 severity and hospitalization outcomes were defined as per categories 4 and 5 of the NIH COVID-19 CTG. Further, the included studies additionally relied on self-reported questionnaires of the participants (study-specific details of ascertainment of SARS-CoV-2 infection or COVID-19 are given in Table 1 [[24], [25], [26], [27], [28], [29], [30], [31], [32], [33], [34], [35], [36], [37], [38], [39]]).

**TABLE 1:** Baseline characteristics of included studies exploring the association between diet and the risk of SARS-CoV-2 infection or COVID-19

Serial number	Author, y; country	Period when study was conducted	Sample size, SARS-CoV-2 infections/COVID-19 cases (%)	Study design, study population	Dietary assessment	Measurement of dietary intake	Outcome of interest	Ascertainment of SARS-CoV-2 infection/COVID-19 disease
**Cross-sectional studies**								
1	Ponzo et al., 2021; Italy [24]	January to February 2021	*N* = 900, *N* = 148 (16.4%)	Observational retrospective study, healthcare professionals	36-food item FFQ	MD adherence measured by Medi-lite ranging 0–18, and its individual components	Risk of SARS-CoV-2 infection	Identified new SARS-CoV-2 infection cases by self-reported questionnaires. A case was confirmed if the participant chose one of the following answers: never, asymptomatic with a positive swab, home-managed infection, infection requiring hospital admission in a non-intensive care unit, or infection requiring the admission to an intensive care unit.
2	Tadbir Vajargah et al. 2022; Iran [25]	June to September 2021	*N* = 250	Observational retrospective study, COVID-19 hospitalized patients	Online validated 168-food item FFQ regarding dietary intake 1 y prior to the COVID-19 diagnosis	Food groups	COVID-19 severity and symptoms	Participants were asked to complete a general questionnaire to obtain information about the presence of each common clinical manifestation of COVID-19 (i.e., fever, rigors, weakness, myalgia, dyspnea, cough, sore throat, nausea, and vomiting). The initial measurement of CRP at hospital admission was obtained from the medical records. NIH CTG classification was used to assess the COVID-19 severity.
3	Zargarzadeh et al., 2022; Iran [26]	June to September 2021	*N*=250	Observational retrospective study, COVID-19 hospitalized patients	Online validated 168-food item FFQ regarding dietary intake 1 y prior to the COVID-19 diagnosis	MD adherence measured by the MedDiet score by Trichopoulou et al. [27], ranging 0–9	COVID-19 severity and symptoms	A general questionnaire to obtain information about the presence of each common clinical manifestation of COVID-19 (i.e., fever, rigors, weakness, myalgia, dyspnea, cough, sore throat, nausea, and vomiting). The initial measurement of CRP at hospital admission was obtained from the medical records. NIH CTG classification was used to assess the COVID-19 severity.
**PROSPECTIVE STUDIES**								
1	Merino et al., 2021; UK and US [28]	March to December 2020	*N* = 592,571, *N* = 31,815 (5.4%)	Prospective cohort study, general population	27-item Leeds Short Form FFQ	Diet quality was measured using the validated healthy plant-based dietary index (hPDI)	Risk and severity of COVID-19	COVID-19 risk defined using a validated symptom-based algorithm to predict whether a participant has been infected with SARS-CoV-2 on the basis of their reported symptoms, age, and sex. COVID-19 severity was ascertained based on a report of the need for a hospital visit which required the following: *1*) noninvasive breathing support, *2*) invasive breathing support, and *3*) administration of antibiotics combined with oxygen support (online Supplementary Methods).
2	Perez-Araluce et al.^1^, 2022; Spain [29]	March to December 2020	*N* = 9485, *N* = 373 (3.9%)	Prospective cohort study, university graduate students in Spain	136-item FFQ	MD adherence measured by the MedDiet score by Trichopoulou et al. [27], ranging 0–9, and its individual food groups	Risk of SARS-CoV-2 infection and risk of COVID-19 and severity	For COVID-19 risk, 2 definitions were used: *1*) participants who reported a positive diagnostic test; *2*) participants with a medical diagnosis or classified as incident cases by the Menni et al. [30] algorithm based on symptoms, age, and sex. For COVID-19 severity: *1*) hospitalization with symptoms compatible with the disease; *2*) symptomatic COVID-19 (cough, cold, respiratory distress, loss of smell or taste,diarrhea, and fever).
3	Perez-Araluce et al.^1^, 2022; Spain [31]	March to December 2020	*N*=9413, *N*=369 (3.9%)	Prospective cohort study, university graduate students in Spain	136-item FFQ	MD adherence measured by the MedDiet score by Trichopoulou et al. [27], ranging 0–9	Risk of SARS-CoV-2 infection	For the incidence of COVID-19, all those who reported a positive result in a SARS-CoV-2 diagnostic test were counted.
4	Deschasaux-Tanguy et al., 2021; France [32]	May to October 2020	*N*=7766, *N*=311 (4.0%)	Prospective web-based cohort of adults	24-h dietary recalls	AHEI-2010 ranging 0–110	Risk ofSARS-CoV-2 infection	To estimate the seroprevalence of SARS-CoV-2 infection at the population level, participants who completed the SAPRIS questionnaires. Volunteer participants received self-sampling dried-blood spot kits by mail between May and October 2020. After processing, serologic analyses were performed using commercial ELISA tests (Euroimmun) to detect anti-SARS-CoV-2 antibodies (IgG) directed against the spike protein S1 domain (ELISA-S). The ELISA-S test was considered positive for values of optical density ratio ≥1.1, indeterminate for values between 0.8 and 1.1, and negative for values <0.8. The main outcome was a positive ELISA-S test. Participants with ELISA-S results in the indeterminate range were excluded from the analyses.
5	Vu et al., 2021^1^; US [33]	March to November 2020	N = 37,988, N = 6482 (17.0%)	Prospective cohort UKB, general population	17-item FFQ	Baseline servings of food groups & components were identified as contributing nutritional factors implicated in immunity were used as study exposures	Risk of SARS-CoV-2 infection	COVID-19 test results from Public Health England have been dynamically linked to the UKB. The regularly updated COVID-19 data table provided to UKB researchers includes participant ID, record date, test location (mouth, nose, throat, trachea, etc.), testing laboratory (71 laboratories listed), and test results (negative or positive). The vast majority of samples tested are from combined nose/throat swabs that are transported in a medium suitable for viruses and subject to PCR testing.^2^
6	Yue et al., 2022; US [34]	2020–2021	*N* = 42,935, *N* = 1941 (4.5%)	Nurses’ Health Study II and Health Professionals Follow-up Study, health professionals.	150-item FFQ	MD adherence measured by the aMED score ranging 0–9; AHEI-2010 score (range: 0–110), The EDIH was developed to assess the insulinemic potential of the whole diet. The EDIP was developed to assess the overall inflammatory potential of the diet.	Risk of SARS-CoV-2 infection and COVID-19 severity	Primary outcome was self-reported SARS-CoV-2 infection, including positive results from an antigen or antibody test. SARS-CoV-2-positive participants were classed into the following 4 categories using a modified WHO clinical progression scale: *1*) asymptomatic; *2*) symptomatic; *3*) independent (persistent cough, sore throat, loss of taste, or loss of smell); symptomatic, assistance needed (shortness of breath or difficulty breathing, fever, muscle aches, or digestive symptoms); and *4*) hospitalization. The secondary outcome was symptomatic SARS-CoV-2 infection derived using a method similar to that of Menni et al. [30] The final prediction algorithm included age and reported COVID-19 symptoms including fever, sore throat, muscle aches, loss of taste, loss of smell, and other symptoms consistent with COVID-19 infection.
7	Sharma et al., 2023; Italy [35]	January to September 2021	*N*=24,325, *N*=1520 (6.2%)	Prospective cohort Moli-sani Study, general population	188-item FFQ	MD adherence measured by the MedDiet score by Trichopoulou et al. [27], ranging 0–9, and its individual food groups	Risk of SARS-CoV-2 infection and COVID-19	Study considered SARS-CoV-2 infection cases if one of the following criteria was met *1*) participants resulted to have anti-NP antibodies; *2*) participants who did not receive vaccine against SARS-CoV-2 at the time of study entry and resulted positive in the anti-spike serum prevalence test performed at study visit; *3*) participants who self-reported to have tested positive for SARS-CoV-2 infection at any testing (e.g., swab, serologic tests) at any time prior to the survey; and *4*) participants who reported to have received a medical diagnosis of COVID-19 any time prior to the survey and therefore to have received therapies (i.e., at home or at the hospital). Self-reported medical diagnosis of COVID-19 was used to identify COVID-19 cases.
**Case-control studies**								
1	El Khoury et al., 2021; Lebanon [36]	Not indicated.	Total *n* of participants = 399, *n* of cases = 150; *n* of controls = 249	Online survey, Lebanese citizens aged 21–64 y without immunocompromising health conditions.	16-item FFQ	MedDiet score by Panagiotakos et al. [37] ranging 0–55 and its individual food groups, and 2 a posteriori-derived dietary patterns.	Risk of COVID-19 and severity	COVID-19 risk was defined as symptomatic COVID-19. The COVID-19 burden was determined on the basis of the number of symptoms and hospitalization as follows; <5 symptoms: mild burden, 5–10 symptoms: moderate burden, >10 symptoms and/or hospitalization: high burden. The hospitalized patients were also classified as nonsevere cases (no disruption of daily life, hindrance to daily life without oxygen requirement, oxygen treatment via nasal canula, and oxygen mask) or severe cases (mechanical ventilation or multiorgan damage and extracorporeal membrane oxygenation).
2	Kim et al., 2021; France, Germany, Italy, Spain, UK, US [38]	July to September 2020.	Total *n* of participants=2884, *n* of cases=568; *n* of controls =2316	Online survey among frontline physicians and nurses	47-item FFQ	Self-reported dietary patterns: *1*) Plant-based diets; *2*) Plant-based diets or pescatarian diets; and *3*) Low-carbohydrate, high-protein diets	Risk of COVID-19 infection, severity and duration of symptoms.	Participants had 5 options: *1*) Very mild: asymptomatic or nearly asymptomatic; *2*) Mild: symptoms (fever <38°C [without treatment], with or without cough, no dyspnea, no gasping, no abnormal imaging findings); *3*) Moderate: (fever, respiratory symptoms, and/or imaging findings of pneumonia); *4*) Severe: meet any of the following— *a*) respiratory distress, respiratory rate ≥30 times/ min; *b*) low oxygen saturation <93% at rest; *c*)PaO_2_/FiO_2_^2^ ≤300 mm Hg; and *5*) Critical: respiratory failure needing mechanical assistance, intensive care unit admission, shock, or extra-pulmonary organ failure.
3	Zamanian et al. 2023; Iran [39]	February to April 2020	Total *n* of participants = 141; *n* of cases =53; *n* of controls =88	COVID-19 hospitalized patients	147-item FFQ	DASH diet score ranging 8–40	Risk of hospitalization due to COVID-19.	The diagnosis was made based on the results of nasopharyngeal swabs for SARS-CoV-2 RT-PCR and chest computed tomography scans. Those who received noninvasive ventilation masks were categorized as the severe group. Patients with COVID-19 infection who were not sick enough to need hospitalization and were treated at home included as the outpatient group. 24 h after patient assignment, 2 physicians independently collected clinical characteristics to increase the accuracy of the collected data.

AHEI-2010, Alternate Healthy Eating Index; aMED, alternative Mediterranean diet; CRP, C-reactive protein; CTG, National Institutes of Health COVID-19 Treatment Guidelines; DASH, Dietary Approaches to Stop Hypertension; EDIH, Empirical Dietary Index for Insulinemia; EDIP, Empirical Dietary Index for Inflammatory Pattern; FiO_2_, fraction of inspired oxygen; FFQ, food frequency questionnaire; MD, Mediterranean diet; NP, nucleocapside protein; PaO_2_, partial pressure of oxygen; SARS-CoV-2, severe acute respiratory syndrome coronavirus 2; UKB, UK Biobank.

1 Study outcome was initially mislabeled as COVID-19 in the study.

2 PaO_2_/FiO_2_ ratio is the *ratio of arterial oxygen partial pressure (PaO2 in mmHg) to fractional inspired oxygen.*

### Selection process

**FIGURE 1:**
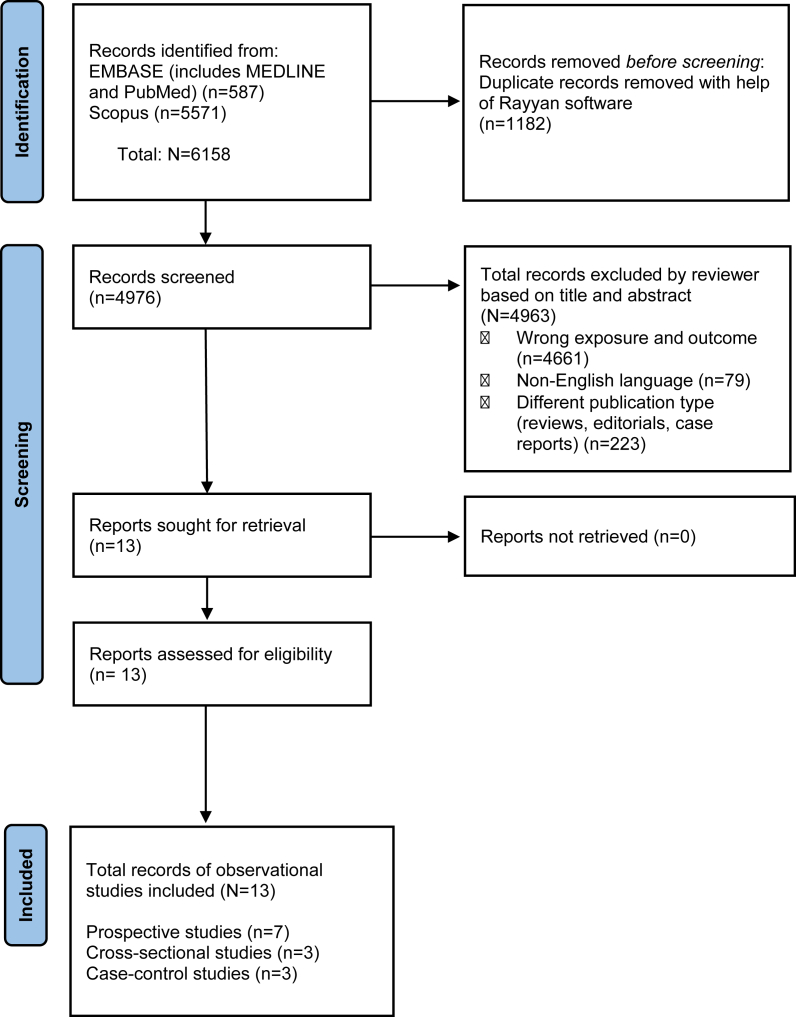
The PRISMA 2020 flow chart shows the study selection process. Identification of studies via databases and registers for diet and risk of SARS-CoV-2 infection or COVID-19.

We used Rayyan Software—an automated web tool [22] for the screening process. The automated tool was used to identify duplicate articles from all the databases. Research articles were screened for potential inclusion based on their title and abstract by 1 reviewer (SS) with help of the “automated screening” feature in the Rayyan Software. Following which, an additional screening conducted by the reviewer (SS) as a precautionary measure to lower selection errors. Finally, the full text versions of the studies were retrieved and evaluated for inclusion based on the inclusion criteria stated above, and were assessed by 2 independent reviewers (SS and MB). The screening process is provided using a PRISMA flow chart, version 2020 (Figure 1) [21].

### Data extraction and data items

The following data were extracted from the included studies: surname of the first author, publication year, country, period when the study was conducted, type of study design, SARS-CoV-2 infection and/or COVID-19 cases, sample size, study population, dietary assessment used for dietary data, measurement of dietary intake, outcome of interest, assessment of SARS-CoV-2 infection and/or COVID-19, covariates, statistical methods, results, and conclusion (TABLE 1, TABLE 2). Two independent reviewers assessed and selected the studies from the data extracted (SS and MB). The data pertaining to the sample, methods, and results from each of the included studies were extracted by 1 author (SS) and was cross-checked by another reviewer (MB). Studies were only included in the review upon mutual agreement of both reviewers (SS and MB) in the data extraction phase.

**TABLE 2:** Results of the included studies exploring the association between diet, and the risk of SARS-CoV-2 infection or COVID-19.

Serial number	Author, y	Outcome of interest	Covariates	Statistical methods	Results	Conclusion
**Cross-sectional studies**						
1	Ponzo et al., 2021 [24]	Risk of SARS-CoV-2 infection	None.	Logistic regression analyses.	The risk of infection was inversely associated with the MeD score (OR: 0.88; 95% CI: 0.81, 0.97 for 1-point increment in the score) and the consumption of cereals (OR: 0.64; 95% CI: 0.45, 0.90).	The risk of SARS-CoV-2 infection was inversely associated with adherence to the MD and cereal intake.
2	Tadbir Vajargah et al., 2022 [25]	COVID-19 severity and symptoms	Age, energy intake, physical activity, and BMI.	Binary logistic regression.	Participants with higher consumption of fruits (OR: 0.28; 95% CI: 0.14, 0.58), vegetables (OR: 0.33; 95% CI: 0.16, 0.69), and dietary fiber (OR: 0.25; 95% CI: 0.12, 0.53) had lower odds of having severe COVID-19.	Higher consumption of fruits, vegetables, and fiber was inversely linked to COVID-19 severity
3	Zargarzadeh et al., 2022 [26]	COVID-19 severity and symptoms	Age, sex, energy intake/BMR, physical activity, dietary supplementation, corticosteroid use, antiviral medication use, and BMI.	Binary logistic regression.	Participants with the highest MD score were 77% less likely to have severe COVID-19 than those with the lowest score (OR: 0.23; 95% CI: 0.11, 0.50 for T3 vs. T1). Higher MD score was inversely associated with COVID-19 symptoms, including dyspnea, cough, fever, chills, weakness, myalgia, nausea and vomiting, and sore throat.	Higher adherence to the MD was associated with a decreased likelihood of COVID-19 severity and symptoms
**Prospective studies**						
1	Merino et al., 2021 [28]	Risk and severity of COVID-19	Age, sex, race/ethnicity, index of multiple deprivation, population density, healthcare worker status, presence of comorbidities (diabetes, cardiovascular disease, lung disease, cancer, kidney disease), BMI, smoking status, and physical activity	Multivariable-adjusted Cox regression models stratified by calendar date at study entry, country of origin, and 10-y age group.	Compared with individuals in the lowest quartile of the diet score, high diet quality was associated with lower risk of COVID-19 (HR: 0.91; 95% CI: 0.88, 0.94) and severe COVID-19 (HR: 0.59; 95% CI: 0.47, 0.74).	A diet characterized by healthy plant-based foods was associated with lower risk and severity of COVID-19.
2	Perez-Araluce et al., 2022 [29]	Risk of SARS-CoV-2 infection and risk of COVID-19 and severity	Age, sex, years of university education, occupation, and marital status, lifestyle habits (smoking status and physical activity), and anthropometric and clinical data (weight, height, and comorbidities).	Multivariable-adjusted logistic regression models.	**SARS-CoV-2 infection risk.** In analyses excluding health professionals (*n* = 6406; *n* of cases = 167), a higher adherence to the MD was linked to a decreased risk of SARS-CoV-2 infection identified as positive diagnostic test (OR: 0.44, 95% CI: 0.22, 0.88) and to lower risk of COVID-19 through medical diagnosis/Menni algorithm [30] (OR: 0.64; 95%CI: 0.42, 0.98). Low consumption of whole dairy products and yogurts were also associated with reduced risk of SARS-CoV-2 infection and COVID-19. In analyses including health professionals (*n*=9485; *n* of cases =373), adherence to MD was not associated with the outcomes. Moderate consumption of fish and low intake of meat were linked to reduced SARS-CoV-2 infection or COVID-19 risks. **COVID-19 severity.** In analyses excluding health professional (*n* of cases = 338), OR for symptomatic COVID-19 associated with high adherence to MD was 0.64 (95% CI: 0.41, 1.00), compared to low adherence. Moderate consumption of whole dairy products was also inversely associated. The MD was not associated with serious COVID-19, whereas moderate/high intakes of legumes were inversely related. In analyses including healthcare professionals (*n* of cases = 565), no associations between dietary factors and the outcomes were observed.	Possible protective effect of the complete MD is more important than that of its individual food groups.
3	Perez-Araluce et al., 2022 [31]	Risk of SARS-CoV-2 infection	Age, sex, years of university education, occupation, and marital status, lifestyle habits (smoking status and physical activity), and anthropometric and clinical data (weight, height, and comorbidities).	Multivariable-adjusted logistic regression models.	Participants with intermediate adherence to the MD (3 < MDS 6) had a significantly lower odds of developing SARS-CoV-2 infection (OR: 0.50, 95% CI: 0.34, 0.73), and those with the highest adherence (MDS > 6) exhibited the lowest risk (OR: 0.36, 95% CI: 0.16, 0.84)	Possible protective effect of the MD.
4	Deschasaux-Tanguy et al., 2021 [32]	Risk of SARS-CoV-2 infection	Sex, age, educational level, employment status, smoking status, presence of children aged <18 y at home, residential area, geographical area, frequency of going out over the past week and prevalent chronic disease (cancer, cardiovascular disease, high blood pressure, diabetes, dyslipidemia), BMI, and physical activity level prior to the March 2020, month of blood draw, the number of 24 h dietary records, intakes of energy (without alcohol), alcohol, and composite index reflecting the adherence to 3 recommended protective behaviors when going out.	Multivariable-adjusted logistic regression models.	No association was observed with overall diet quality as measured by the AHEI-2010 (OR: 0.96; 95% CI: 0.85, 1.09); or by the Programme National Nutrition Santé-guidelines score 2 (OR: 0.95; 95% CI: 0.82, 1.10), or with the proportion of ultraprocessed food in the diet (OR: 0.96; 95% CI: 0.85,1.08) Dietary fibers (OR: 0.84; 95% CI: 0.72, 0.98) and fruit and vegetables (OR: 0.85; 95% CI: 0.74, 0.97) were associated to a decreased probability of SARS-CoV-2 infection whereas dairy products (OR: 1.19; 95%CI: 1.06, 1.33) were associated to increased odds.	No association was observed with overall diet quality and risk of SARS-CoV-2 infection. Higher dietary intakes of fruit and vegetables and fibers were associated with a lower susceptibility to infection.
5	Vu et al., 2021 [33]	Risk of SARS-CoV-2 infection	Age, sex, race, education, employment status, type of accommodation lived in and number of cohabitants, smoking behaviors, current health status, socioeconomic status index, BMI, physical activity, comorbidities, such as diabetes, history of any heart diseases, and hypercholesterolemia or hypertensive medication.	Multivariable-adjusted logistic regression models.	OR for SARS-CoV-2 infection risk was 0.90 (95% CI: 0.83, 0.96) when consuming 2–3 cups of coffee/d (vs. <1 cup/d); OR, 0.88 (95% CI: 0.80, 0.98) when consuming vegetables in the third quartile of servings/day (vs. lowest quartile); OR, 1.14 (95% CI: 1.01, 1.29) when consuming fourth quartile servings of processed meats (vs. lowest quartile), and OR: 0.91 (95% CI: 0.85, 0.98) when having been breastfed (vs. not breastfed)	Consumption of coffee, vegetables, and being breastfed as an infant were inversely associated with incident SARS-CoV-2 infection; intake of processed meat was directly associated.
6	Yue et al., 2022 [34]	Risk of SARS-CoV-2 infection and COVID-19 severity	Age, sex, and race, smoking, physical activity, energy intake, census tract median family income, census tract median family home value, census tract population density, concern about COVID-19, interaction with people other than patients with presumed or documented COVID-19 and frontline health care providers and personal protective equipment use, BMI, history of high cholesterol, history of high blood pressure, and presence of other pre-existing medical conditions.	Multivariable-adjusted logistic regression models.	Healthier diets, represented by higher AHEI-2010 and aMED scores and lower EDIH and EDIP scores, were associated with lower likelihood of SARS-CoV-2 infection (Q4 vs. Q1: OR: 0.80; 95% CI: 0.69, 0.92 for AHEI-2010; OR: 0.78; 95% CI: 0.67, 0.92 for aMED; OR: 1.36; 95% CI: 1.16, 1.57 for EDIH; and OR: 1.13; 95% CI: 0.99, 1.30 for EDIP). Higher adherence to the AHEI and aMED and lower adherence to the EDIP and EDIH were associated with lower likelihood of severe infection. Participants with 1 SD higher scores of the AHEI-2010 and aMED were 20%–22% less likely to be hospitalized owing to SARS-CoV-2 infection. Participants with 1 SD higher scores of the EDIH and EDIP had a 23%–37% higher likelihood of hospitalization.	Healthier diets, represented by higher AHEI-2010, aMED, EDIH, and EDIP scores, were associated with lower likelihood of SARS-CoV-2 infection or severity. Participants with healthier diet had lower likelihood of severe infection and were less likely to be hospitalized owing to COVID-19.
7	Sharma et al., 2023 [35]	Risk of SARS-CoV-2 infection and COVID-19	Age, sex, energy intake, educational level, occupational class, marital status, being health professional, smoking status, BMI, leisure-time physical activity, baseline history of CVD, cancer, diabetes, hypertension, dyslipidemia, number of chronic diseases diagnosed since March 2020, and the composite index of behavioral and environmental risk factors for SARS-CoV-2 infection.	Multivariable-adjusted logistic regression models.	The Mediterranean Diet Score was not associated with the likelihood of SARS-CoV-2 infection (OR: 0.94; 95% CI: 0.83, 1.06) or COVID-19 (OR: 0.82; 95% CI: 0.62, 1.10) diagnosis. High consumption of cereals was associated with lower odds of SARS-CoV-2 infection (OR: 0.91; 95% CI: 0.83, 1.00; for each 25 g/d increase). Likelihood of having been diagnosed with COVID-19 decreased in association with increasing olive oil intake (OR: 0.10; 95% CI: 0.01, 0.79; for each additional 10 g/d), moderate alcohol consumption (OR: 0.18; 95% CI: 0.04, 0.82) and higher intakes of fruits and nuts (OR: 0.89; 95% CI: 0.79, 0.99).	No significant protective effect of a Mediterranean diet. Among food groups, higher intake of cereal was associated with lower odds of SARS-CoV-2 infection, whereas olive oil, moderate alcohol intake, fruits and nuts were linked to reduced COVID-19 risk.
**Case-control studies**						
1	El Khoury et al., 2021 [36]	Risk of COVID-19	Age, pre-existing health conditions, and respiratory diseases.	Multiple multinominal logistic regression tests.	Adherence to MD was inversely associated with COVID-19 (OR of not being infected by COVID-19: 1.055; 95% CI: 1.013, 1.099). No association with Western dietary pattern. Risk of COVID-19 burden was not associated with the MedDiet score (OR: 0.946; 95% CI: 0.832, 1.077) nor with a Western dietary pattern (OR: 0.384; 95% CI: 0.097, 1.528 compared with a Prudent diet).	MD was associated with lower risk of COVID-19 but not with burden.
2	Kim et al., 2021 [38]	Risk of COVID-19 infection, severity and duration of symptoms.	Age, sex, race/ethnicity, and country, medical specialty, smoking status, and physical activity, BMI, and presence of medical conditions.	Multivariable-adjusted logistic regression models.	**Risk of moderate-to-severe COVID-19.** Compared to those not following these diets, “plant-based diets” (OR: 0.28; 95% CI: 0.10, 0.82) and “plant-based or pescatarian diets” (OR: 0.41; 95% CI: 0.16, 0.99) were associated with lower risk. “Low-carbohydrate, high-protein diets” tended to be associated with increased risk (OR: 1.47; 95%CI: 0.88, 2.47). Compared with participants who reported following “plant-based diets,” those who reported following “low-carbohydrate, high-protein diets” had greater odds of moderate-to-severe COVID-19 (OR: 3.96; 95% CI: 1.14, 13.75). **COVID-19 risk and duration.** No association between self-reported diets with risk or duration of COVID-19.	Plant-based diets or a spectrum of plant-based diets were associated with lower odds of moderate-to-severe COVID-19. Low-carbohydrate, high-protein diets were associated with greater odds of moderate-to-severe COVID-19 illness, compared with individuals following plant-based diets.
3	Zamanian et al., 2023 [39]	Risk of COVID-19 related hospitalization	Sex, age, BMI, and daily energy intake	Multivariable-adjusted logistic regression models.	The risk of hospitalization in the highest tertile of DASH score was 81% lower than the lowest tertile (OR: 0.19; 95% CI: 0.07, 0.55).	Higher adherence to a DASH diet lowered the risk of hospitalization due to COVID-19.

AHEI-2010, Alternate Healthy Eating Index; aMED, alternate Mediterranean diet; BMR, basal metabolic rate; CI, confidence interval; CVD, cardiovascular disease; DASH, Dietary Approaches to Stop Hypertension; EDIH, Empirical Dietary Index for Hyperinsulinemia; EDIP, Empirical Dietary Inflammatory Pattern; MD, Mediterranean diet; OR, odds ratio; SARS-CoV-2, severe acute respiratory syndrome coronavirus 2.

### Statistical analysis

Due to the heterogeneity of the exposure and outcome measurements, and the small number of included studies in this systematic review that have reported the association between diet, and SARS-CoV-2 infection or COVID-19, the data extracted were deemed to be unsuitable for a meta-analysis. Thus, a systematic review was conducted for the same.

### Quality assessment process

The National Heart, Lung and Blood Institute (NIH) Quality Assessment Tools [40] for observational cohort and cross-sectional studies (14-item criteria) and case-control studies (12-item criteria) were used to assess the risk of bias of the included studies. This tool does not use a points system to generate an overall quality of assessment score for the included studies. Rather, the categories for methodological quality are based on an overall judgment of the study: “Poor,” “Fair,” and “Good.” Two independent evaluators assessed the quality of the included studies, and any disagreements were resolved through discussing the relevant parts of the paper to check if they had misinterpreted any element. Evaluators (SS and MB) had to select “Yes,” “No,” or “Not Reported/Not Applicable/Unable to Determine” in the NIH tool. An overall assessment for each study was generated based on the number of times “Yes” was selected under each criterion of the NIH tool; a “Good” study had a maximum of 3 categories that were not rated as a “Yes.” Two categories, “validity of outcomes” and “adjustment of confounders,” were considered as most important criteria to determine the classification of study quality.

Results of the quality assessments of individual studies for SARS-CoV-2 infection or COVID-19 are summarized in Supplementary Table 1A–C**.**

## Results

### Literature selection

#### Diet and the risk of SARS-CoV-2 infection or COVID-19

A total of 6158 studies were identified from EMBASE (MEDLINE and PubMed) and Scopus databases that explored the relationship between diet and the risk of SARS-CoV-2 infection or COVID-19. An automated tool, Rayyan Software, was used to eliminate the duplicate records. The remaining 4976 records were screened for their eligibility based on title and abstract, of which 4963 studies were excluded for the reasons cited in the PRISMA flow chart in Figure 1. The full text versions of the remaining 13 studies were retrieved and assessed for eligibility. Therefore, a total of 13 observational studies were included in this systematic review.

### Characteristics of the included studies

In total, 13 studies were included in the review for the association between diet quality and: *1*) risk of SARS-CoV-2 infection (4 studies) [24,[31], [32], [33]]; *2*) COVID-19 (6 studies) [25,26,28,36,38,39]; *3*) both outcomes (3 studies) [29,34,35]. Of which, 3 were case-control [36,38,39] and 3 cross-sectional [[24], [25], [26]], and 7 studies had a prospective study design [28,29,[31], [32], [33], [34], [35]] and were published between February 2020 to June 2023 (for further details, refer to Table 1).

It should be noted that few early studies [29,31,33] mislabeled the study outcome: SARS-CoV-2 infection as COVID-19; for this, we critically appraised the study methodology and reclassified the study outcome for better clarity (refer to TABLE 1, TABLE 2).

Among the cross-sectional studies, 2 were conducted in Iran [25,26] and one in Italy [24]. The sample sizes ranged from 250 to 900 participants. The Italian study participants were health care professionals, whereas the Iranian studies included patients hospitalized for COVID-19. The Italian [24] and one Iranian [26] studies explored the Mediterranean diet as dietary exposure [26], whereas the other Iranian study examined individual food groups as the study exposure [25]. Further, the Italian study utilized a 36-food item food frequency questionnaire (FFQ) to record dietary data, whereas the Iranian studies used an online validated 168-food item FFQ. The outcomes among these 3 studies differed; the Italian study explored the risk of SARS-CoV-2 infection, but the Iranian studies explored the risk of COVID-19 severity and symptoms.

Among the prospective studies, 3 of them were from United States and United Kingdom [28,33,34], 2 from Spain [29,31], and 1 each from France [36] and Italy [35]. The sample size of these 5 cohorts ranged between 1520 and 592,571 participants, mostly recruited from the general population, university graduate students, or health professionals. Dietary data were recorded using varied methods: a couple of studies used concise versions of FFQ, a 17-food item FFQ [33] and a 27-food item FFQ [28]; whereas one study from France used a 24-h dietary recall [32], and the rest of the studies used a detailed FFQ [29,31,34,35]. All 7 studies computed and explored the dietary exposure in varied formats. 3 studies assessed adherence to Mediterranean diet through the score proposed by Trichopoulou [27] (ranging 0–9) and its individual food components [29,31,35], and one study used the alternate Mediterranean diet (aMED) adherence score ranging from 0 to 9 [34]. Whereas, 1 study analyzed only food components [33], and 3 studies used validated healthy plant-based diet index (hPDI) score [28] and the alternate healthy eating index score (AHEI-2010) [32,34].

Case-control studies explored risk of COVID-19 but examined different exposures: MedDiet score (0–55 points) and a *posteriori*-derived dietary patterns [36], and self-reported dietary patterns [38,39] (refer to Table 1 for further study details). The sample size ranged between 141 and 2884 participants, mostly recruited from online surveys. Dietary data was obtained by using concise versions of FFQ in 2 studies (16- or 47-item FFQs) [36,38], whereas the Iranian study relied on the use of a 147-item FFQ [39].

### Results of the included studies

Most studies included in the systematic review reported an inverse association between diet quality and the risk of SARS-CoV-2 infection or COVID-19 risk or severity, regardless of their study design (refer to Table 2 for detailed results).

The Mediterranean diet was examined as dietary exposure in 7 studies. An inverse risk of SARS-CoV-2 infection associated with a Mediterranean diet was reported in 1 cross-sectional (odds ratio [OR]: 0.88; 95% confidence interval [CI]: 0.81, 0.97 for each 1-point increment in the dietary score) [24], and 1 prospective study [34] (OR: 0.78; 95% CI: 0.67, 0.92 for highest compared with lowest quartile of adherence to aMED). Participants with high adherence to a Mediterranean diet experienced lower risk of SARS-CoV-2 infection or COVID-19 in 2 prospective studies conducted among the SUN cohort of Spanish graduates [29,31]. A longitudinal analysis from the Moli-sani Study in Italy [35] showed an inverse association with both risk of SARS-CoV-2 infection (OR: 0.94; 95% CI: 0.83, 1.06 for 1-unit increase in the Mediterranean Diet Score) or COVID-19 (OR: 0.82; 95% CI: 0.62, 1.10), although statistical significance was not retained. A case-control study on Lebanese participants reported an inverse association between a Mediterranean diet and risk of COVID-19 but not with COVID-19 severity [36].

However, a Mediterranean diet was inversely associated with COVID-19 severity in 1 cross-sectional study (OR: 0.23; 95% CI: 0.11, 0.50 for T3 vs. T1) [26], and in prospective analyses on US adults (OR for 1 SD increment in the aMED score: 0.87; 95% CI: 0.76, 0.99 and OR: 0.88; 95% CI: 0.82, 0.94) for risk of independent symptoms and assistant-needed symptoms, respectively) [34], but not in the SUN cohort (OR: 0.30; 95% CI: 0.03, 2.57) [29].

A DASH diet was inversely related to COVID-19 hospitalization (OR: 0.19; 95% CI: 0.07, 0.55) in 1 case-control study [39], whereas a healthy plant-based diet had an inverse association with both COVID-19 infection (HR: 0.91; 95% CI: 0.88, 0.94) and severity (HR: 0.59; 95%CI, 0.47, 0.74) [28].

The AHEI-2010 was associated with lower risk of SARS-CoV-2 infection in US adults (OR: 0.80; 95% CI: 0.69, 0.92 for highest vs. lowest quartile of the score), and COVID-19 severity (OR for 1 SD increment: 0.85; 95% CI: 0.80, 0.90 for assistant-needed symptoms outcome), although it was not associated with SARS-CoV-2 infection among participants from the French Nutrinet-Santè cohort (OR: 0.96; 95% CI: 0.85, 1.09) [32,34].

A hyperinsulinemic diet was found associated with increased risk of both SARS-CoV-2 risk (OR: 1.36; 95% CI: 1.16, 1.57 for highest vs. lowest quartile) and COVID-19 severity; a proinflammatory diet was directly linked to COVID-19 severity (OR for 1 SD increment: 1.09; 95% CI: 1.02, 1.15 for assistant-needed symptoms outcome) [34].

Among studies that explored only individual food components as the exposure [26,33], it was observed that a higher consumption of fruits, vegetables, and dietary fiber was associated with lower risk of SARS-CoV-2 infection or COVID-19 (refer to Table 2). All studies analyzed data using multivariable-adjusted models, except one [24]. Most studies adjusted the models for age, sex, BMI, comorbidities, and physical activity level.

### Quality assessment findings

The NIH tool was used to assess the risk of bias of the studies that were included in the review (refer to Supplementary Table 1A–C for a summary of the risk of bias assessment).

Six studies (1 cross-sectional, 1 case-control, and 4 prospective studies) scored “Good” overall, deeming them to be at low risk of bias. However, 7 studies (2 each of case-control and cross-sectional studies and 3 prospective studies) scored “Fair” overall, deeming them to have a slightly higher risk of bias due to the following reasons: definition, selection of key confounders, reliability and consistent implementation of measurement of exposures, and assessment duration of exposures.

## Discussion

The aim of this systematic review was to examine the evidence from observational studies evaluating the association between diet and the risk of SARS-CoV-2 infection or COVID-19. The overall results, based on 13 studies, indicated that a higher adherence to healthy nutritious diets was inversely associated with the risk of SARS-CoV-2 infection or COVID-19. The studies included in the systematic review underpinned a gap in the literature on the requirement to assess the association of the dietary impact on the risk of SARS-CoV-2 infection or COVID-19 among observational studies.

Previous studies extensively explored the impact of contracting SARS-CoV-2 infection or COVID-19 on diet or the dietary habits/dietary quality during the pandemic and reported a modification in the lifestyle, and diet quality [41,42]. Whereas, few studies focused on short-term effects of isolated foods in the form of functional foods, for example, garlic, ginger, flax seeds, and dragonfruit [[14], [15], [16]], and single nutrients, for example, vitamin D [43,44], vitamin C, zinc, and selenium [19,20,45], on the risk of SARS-CoV-2 infection or COVID-19. This seemed to indicate an inadequate reductionist approach of exploring the effects of single nutrients or food items on a disease of interest [[46], [47], [48]] because foods are mostly consumed in various combinations, making it difficult to attribute the therapeutic effect to a single nutrient or food item of interest [[46], [47], [48]]. Since the examination of dietary patterns is beneficial in the formation of dietary guidelines [46], our decision to examine the associations between the diet (exposure) in the form of dietary patterns, food components or index scores remained justified.

To the best of our knowledge, this is the first systematic review that investigated the association between diet quality and SARS-CoV-2 infection or COVID-19 among observational studies, thus, aiming to capture an entire SARS-CoV-2 infection and disease spectrum, i.e., from contracting the infection to hospitalization due to COVID-19. A systematic review explored the effect of Mediterranean diet on the inflammatory biomarkers among overweight/obese adults from randomized controlled trials and case-control studies, and observed that a hypocaloric, fiber-dense Mediterranean diet could help lower the inflammatory markers among a high BMI adult population at risk of developing COVID-19 [49]. Whereas, our systematic review examined diet quality across all available observational studies (dietary patterns and food component intakes) recorded prior and/or during the COVID-19 pandemic, and explored associations with the risk of SARS-CoV-2 infection or COVID-19.

The results of the included studies were conducted in different countries, i.e., Italy, Spain, United Kingdom, Iran, United States, Lebanon, and France, making the results widely generalizable and replicable. Although the studies measured the dietary data in different formats, for example, Mediterranean diet [24,26,29,31,[34], [35], [36]], AHEI-2010 [28,32], and food groups and individual components [25,33], the results of all the studies consistently suggested that a nutritious diet might be beneficial in lowering the risk of SARS-CoV-2 infection or COVID-19, despite differing in covariate adjustment choices.

Dietary assessment tools and the techniques used to record the dietary data varied among all the studies. Among studies that used FFQ as the dietary assessment tool, the study by Merino et al. [28] used a validated symptom-based algorithm to identify the COVID-19 cases across different populations as their study design included web-based participant recruitment. However, the methodology to compute the diet scores was lesser in detail as they used an FFQ (28-food item Leeds FFQ). This might have caused an underrepresentation of food components and intake important to compute the diet score index. Whereas few studies [26,29,34,35,39] used a detailed FFQ to record the dietary data of the participants in their study.

Further, these studies used the SARS-CoV-2 infection diagnostic results as a confirmation—which might have introduced misdiagnosis and measurement errors in identifying the degree of risk of COVID-19. However, the definition of SARS-CoV-2 infection and COVID-19 was not as detailed as the study by Zargarzadeh et al. [26] and Sharma et al. [35], but, was more robust than in the Merino et al. [28] and the Vu et al. [33] studies, respectively. In contrast, the Zargarzadeh et al. [26] study used the initial measurement of C-reactive protein (CRP) at hospital admission, obtained from the medical records, as well as the 5 levels of severity prescribed by the NIH CTG, whereas Sharma et al. [35] used participant data of anti-nucleocapside protein antibodies for SARS-CoV-2 infection diagnosis in their cohort study.

Further, the study by Vu et al. [33] in the UK Biobank cohort also used an FFQ (17-food item) to record the dietary data similar to the Merino et al. [28] study. This might have affected the quality of the dietary intake assessment, but, nevertheless, the UK Biobank cohort was linked to the national health registry with available COVID-19 data.

In contrast to the studies that used the FFQ as a dietary assessment tool, the Deschasaux-Tanguy et al. [32] study used a 24-h dietary recall to compute the AHEI-2010 score, and utilized a robust methodology to ascertain the SARS-CoV-2 infection but not COVID-19 by using commercial enzyme-linked immunosorbent assay (ELISA).

Although effective hygiene measures, including sanitizers, handwash using soap, K95 masks, and social distancing mandates curbed the overall SARS-CoV-2 infection and COVID-19 rates, studies suggested that infection contraction was related to immunity status, and that COVID-19 was observed among people with low immunity levels [[50], [51], [52], [53]]. Individuals with low grade chronic inflammation have a poor innate immune system, which increases their likelihood of infection [11,54], besides other factors, including genetics, BMI, physical fitness, vaccination status, gut microbiota, stress, illness (cardiovascular disease, diabetes mellitus, cancer, arthritis, obesity, and inflammatory bowel diseases), and diet (nutritional status) [4]. Among these, evidence suggested that optimal nutritional status and better diet quality adherence could potentially be associated with lower risk of contracting the SARS-CoV-2 infection and subsequent COVID-19 [28,35,55,56]. Studies have suggested that a diet high in olive oil, cereals, fruits and nuts, and vegetables were associated with lower odds of SARS-CoV-2 infection and COVID-19. Underlying biological plausibility explaining these associations include anti-inflammatory markers, including IL-6, CRP, antioxidants, antithrombotic effects, and adhesion factors that are beneficial in COVID-19 prevention [28,32,35,38]. Therefore, promotion of a better diet quality to lower the risk of SARS-CoV-2 infection is vital to decrease the subsequent risk of COVID-19. This is because SARS-CoV-2 infection causes angiotensin-converting enzyme (ACE) and its homolog angiotensin-converting enzyme 2 (ACE/ACE2) balance disruption and renin-angiotensin-aldosterone system (RAAS) activation, which ultimately leads to COVID-19 progression. This is especially seen among individuals with comorbidities, such as diabetes mellitus, hypertension, and cardiovascular disease, which are also preventable through healthy dietary interventions [[57], [58], [59], [60]]. Therefore, a dual approach could potentially be a healthy diet quality adherence impacting overall immunity levels coupled with effective hygiene measures, thus lowering SARS-CoV-2 infection, and eventually COVID-19.

### Strengths and limitations

Our study had some strengths and weaknesses to be considered. Despite a relatively low number of observational studies included in the systematic review, NIH risk of bias tool and quality assessment tool yielded an overall score of “Fair.” The NIH tool was selected because it was simple yet robust, replicable, and widely used in systematic reviews related to diet and disease [[61], [62], [63], [64], [65]]. The NIH tool was user-friendly with a concise set of questions focusing on each criterion that directly impacted overall quality assessment ratings, and did not use a points system for assessment but was based on the judgment of study. It was possible to discriminate and compare the responses for each criterion within each study and the corresponding ratings and between multiple studies (overall quality assessment of each study). Further, the tool helps identify and evaluate potential flaws in study methodologies, including bias (for example, participant selection), measurement and selection of key confounders, inclusion and exclusion criteria definition, assessment of exposure and outcome measurements, and other criteria. Finally, the NIH tool provided brief guidance for each question/criterion helpful for evaluators to efficiently conduct quality assessments—lowering overall risk of bias and judgment error while conducting large systematic reviews.

We included all available observational studies that computed the dietary data in varied measurement formats, but underreporting and bias must be considered as the dietary data was self-reported [66,67]. Non-English articles (*n* = 79) were excluded in the screening phase, but these studies did not examine this review’s objective.

This systematic review screened data from multiple databases and used an efficient automated tool, Rayyan Software, to manage the publications, screening, and data extraction process compared to conventional methods, for example, Excel sheets—introducing potential bias and errors in the methodology.

It should also be noted that our systematic review focused on diet quality as the exposure, and not on exposures, such as food habits or ultraprocessed foods. Additionally, this systematic review presents evidence for the entire SARS-CoV-2 infection spectrum potentially contributing toward future research and public health policies. However, few studies, especially those conducted in the early stages of the COVID-19 pandemic, did not adequately define the COVID-19 outcome—because some studies actually analyzed SARS-CoV-2 infection and not COVID-19 per se.

## Conclusion

The overall findings of the observational studies included in this review consistently suggested that a nutritious diet might lower the risk of SARS-CoV-2 infection or COVID-19. These results elucidate the importance of a wholesome diet that is beneficial in potentially protecting against SARS-CoV-2 infection or COVID-19. This systematic review highlights the limited number of observational studies, which could be of public health importance and for cautious nutritional advice in clinical settings.

### Author contributions

The authors’ responsibilities were as follows – MB, SS: designed the research; SS: conducted the systematic literature search, performed the quality assessment and the data extraction; SS, MB: assessed the data extracted and selected the studies for inclusion after independent assessment, and mutual agreement as applicable, and conducted the quality assessment of the studies; SS: wrote the manuscript; MB, ADC, CC, MBD, GdG, LI: critically reviewed the manuscript; and all authors: read and approved the final manuscript.

### Conflict of interest

LI reports financial support was provided by a grant of the Umberto Veronesi Foundation (Bando Covid-19, linea 1, Research project ‘Burden of SARS-CoV-2 infection in populations with high or low risk of infection’). All other authors report no conflicts of interest.

### Funding

The present analyses were partially supported by the Italian Ministry of Health (Ricerca Corrente 2022-2024) and the Fondazione Umberto Veronesi (Bando Covid-19, linea 1, Research project ‘Burden of SARS-CoV-2 infection in populations with high or low risk of infection’). SS was supported by the Joint Platform Umberto Veronesi Foundation-Department of Epidemiology and Prevention at IRCCS Neuromed in Pozzilli, Italy. The funders had no role in study design, collection, analysis, and interpretation of data, nor in the writing of the manuscript or in the decision to submit the article for publication. All authors were and are independent from funders.
